# Can daily actigraphic profiles distinguish between different mood states in inpatients with bipolar disorder? An observational study

**DOI:** 10.3389/fpsyt.2023.1145964

**Published:** 2023-06-09

**Authors:** Yinlin Zhang, Xinyi Deng, Xueqian Wang, Huirong Luo, Xu Lei, Qinghua Luo

**Affiliations:** ^1^Department of Psychiatry, The First Affiliated Hospital of Chongqing Medical University, Chongqing, China; ^2^Sleep and Neuroimaging Center, Faculty of Psychology, Southwest University, Chongqing, China; ^3^Key Laboratory of Cognition and Personality, Ministry of Education, Southwest University, Chongqing, China

**Keywords:** bipolar disorder, actigraphy, motor activity, Mania, depression, mixed-state

## Abstract

**Background:**

Criterion A changes for bipolar disorder (BD) in the Diagnostic and Statistical Manual of Mental Disorders–Fifth Edition yield new difficulties in diagnosis. Actigraphy has been used to capture the activity features of patients with BD. However, it remains unclear whether long-term actigraphic data could distinguish between different mood states in hospitalized patients with BD.

**Methods:**

In this observational study, 30 hospitalized patients with BD were included. Wrist-worn actigraphs were used to monitor motor activity. The patients were divided into bipolar disorder–depression (BD-D), bipolar disorder–mania (BD-M), and bipolar disorder–mixed state (BD-MS) groups. Motor activity differences were estimated using non-parametric analyses between and within the three groups.

**Results:**

The mean 24 h activity level differed between the groups. In the between-group analysis, the intra-individual fluctuation and minute-to-minute variability in the morning and the mean activity level and minute-to-minute variability in the evening significantly differed between the BD-M and BD-MS groups. In the within-group analysis, the BD-M group showed a disrupted rhythm and reduced activity complexity at night. Both the BD-D and BD-MS groups demonstrated significant differences between several parameters obtained in the morning and evening.

**Conclusion:**

The mean activity levels during the relatively long monitoring period and the intra-day variation within the groups could reflect the differences in motor activity. Sustained activity monitoring may clarify the emotional states and provide information for clinical diagnosis.

## Introduction

Bipolar disorder (BD) is a chronic and disabling psychiatric disorder characterized by frequent and repeated depressive and hypomanic/manic episodes (1). The current treatment for BD aims to prevent subsequent episodes, remit symptoms, and enable patients to regain a normal quality of life (2). However, even with treatment, 19–25% of patients experience recurrence annually (3). An increased frequency of previous episodes is associated with a higher risk of recurrence (4). Patients with BD are also more susceptible to changes in the homeostatic network, as shifts in mood are accompanied by changes in energy, activity, behavior, and sleep (5, 6).

The Diagnostic and Statistical Manual of Mental Disorders–Fifth Edition (DSM-5) added an increase in energy and activity, alongside mood changes, to criterion A for hypomania/mania (7). Like the DSM-5, the International Classification of Diseases (ICD)–11th revision included increased psychomotor disturbances (activity) with mood changes in criterion A (8). This modification can prevent overdiagnosis of BD but at the cost of a possible delay in the detection of hypomanic/manic visits, thus making relapses of hypomanic/manic episodes less recognizable (9, 10). Therefore, more clinical evidence is needed to better identify various mood states. Different activity patterns can indicate the mood states of patients (11). Related actigraphy studies have confirmed that patients with bipolar disorder–mania (BD-M) showed higher average daily activity levels and greater complexity and variability during active morning periods than did patients with bipolar disorder–depression (BD-D) (12). Gonzalez et al. investigated the association between activity rhythmicity and mood state in patients with BD and found that more severe manic symptoms were associated with more disrupted activity patterns (13). Some meta-analytic evidence suggests that further study of activity patterns in patients with BD may help in the identification of different mood states and supplement traditional diagnosis (14).

Owing to its simplicity and objectivity, actigraphy has recently been used extensively in the evaluation of severe mental illnesses (15, 16). As a new technology, actigraphy has been recognized for its validity in measuring the motor activity and sleep patterns of patients with BD (17–19). Changes in the motor activity measured on digital actigraphy may indicate mood states. Actigraphy captures the time-varying features of BD and is often used to evaluate sleep data and daytime activity separately (14). Previous research on the variability of activity patterns within 24 h showed no difference in the median levels of activity in admitted patients with BD but markedly different patterns during a consecutive 64-min morning activity between hospitalized patients with BD-M and BD-D (11). However, few studies have focused on the relatively long-term motor activity of patients with BD with different mood states, including bipolar disorder–mixed state (BD-MS). Our study aimed to explore whether a relatively long actigraphy monitoring of acute episodes can help identify psychomotor disturbances in patients with different mood states of BD and provide information for BD diagnosis. The following research questions were raised:

- Are motor activity patterns beyond 24 h meaningful in the identification of the different mood states of patients with BD?
- What is the extent of variation in the motor activity of patients with different mood states of BD?

## Materials and methods

### Study overview and procedure

This study had a mixed design and investigated the influence of three mood states of inpatients with BD—BD-M, BD-D, and BD-MS—on motor activity during a relatively long objective monitoring. Participants underwent The Mini-International Neuropsychiatric Interview (MINI), a short Structured Clinical Interview (20). All diagnoses were made in accordance with the ICD-10 Criteria for Research (21). All participants voluntarily participated in the study after acknowledging the detailed study information. Patients who met the inclusion criteria completed a demographic assessment and questionnaire, which assessed their current mood symptom levels. Each patient wore a watch-based actigraph from the earliest stage of hospitalization (within the first 3 days of admission) to the time of discharge. Valid actigraphic data obtained within 3–10 consecutive days were included in the analysis.

### Ethics and consent

All procedures in this work were performed in compliance with the ethical standards of relevant national and institutional committees on human experimentation and the Helsinki Declaration of 1975, as revised in 2008. The ethical committee of the First Affiliated Hospital of Chongqing Medical University approved the study (No. 2020-K10). The study received support from the Chongqing Medical Scientific Research Project (Joint Project of Chongqing Health Commission and Science and Technology Bureau; No. 2020GDRC026) and the National Key Research and Development Program of China (No. 2021YFC2501500). All participants provided written informed consent. For adolescents aged under 18 years, we obtained written informed consent from their legal guardians. The capacity to consent was established by a senior psychiatrist, and written consent was recorded.

### Participants

The participants included inpatients aged 14–55 years with a confirmed diagnosis of BD admitted to the Department of Psychiatry of the First Affiliated Hospital of Chongqing Medical University, China, between February 14, 2022, and July 15, 2022. All diagnoses were made by three psychiatrists, of whom at least one was responsible for the clinical treatment of the patients. The inclusion criteria were as follows: (1) diagnosis of BD, (2) depression, mania, or mixed state of BD, and (3) normal daily activity performance. The exclusion criteria were as follows: (1) known medical conditions that severely impact daily activity performance (e.g., history of traumatic head injury), (2) alcohol or psychoactive substance abuse, (3) pregnancy or lactation, and (4) non-cooperative state needing constant restraint.

A total of 34 participants were included in the study. The diagnostic groups included the BD-M, BD-D, and BD-MS groups (F31.1–F31.6). Among the 34 participants, four were excluded because their activity data were obtained within <3 days only. The main reasons for the short wearing time were forgetting to wear the actigraph after showering or taking it off because of an uncomfortable wearing experience.

### Measurement and recording

#### Clinical assessments

The demographic assessment included age, sex, and years of education. A history of self-injury and suicidal ideation was recorded. At the beginning of the study, independent researchers assessed the depressive symptoms of participants using the 17-item Hamilton Depression Scale (HRSD-17) (22) both in adults and adolescents. The validity and reliability of the Chinese version of HRSD-17 have been verified (23). And the manic/hypomanic symptoms were evaluated using the Young Mania Rating Scale (YMRS) (24, 25). The HRSD-17 scores ranged from 0 to 52. Depressive symptoms (5) were categorized as follows: not experiencing depression (defined as HRSD-17 score ≤ 7), mild (score 8–13), moderate (score 14–19) and severe (score ≥ 20). Higher score of YMRS indicated a higher level of mania/hypomania. Manic symptoms (5) were classified as not experiencing mania (defined as a YMRS score ≤ 6), mild (score 7–14), moderate (score 15–19) and severe (score ≥ 20).

#### Actigraphy

High-resolution motor activity was measured using the wrist-worn actigraph (wGT3X-BT, version 1.9.2, ActiGraph LLC) at 1-min intervals. The collected raw data were stored directly in a non-volatile flash memory device and converted into a variety of objective activity data using publicly available algorithms. Data download, observation, and processing were completed using the actigraph software program ActiLife (version 6.13.3). Data collection began at 6:00 p.m.; the morning period was defined from 6 a.m. to 3 p.m. and the evening period from 3 p.m. to 12 p.m. (11, 26–28). Considering the difference in the length of hospitalization, we calculated the activity counts per minute for 30 participants (266 days in total) whose valid data lasted over 3 days and excluded the data exceeding 10 days.

#### Activity parameters

We analyzed the mean activity counts per minute for the entire 24 h recording period for 3–10 consecutive days. The activity parameters included the following (26, 27):

- Mean activity counts per minute as the measurement of overall activity levels;
- Standard deviation (SD)/min in % of the mean as the measurement of intra-individual fluctuations of activity;
- Root mean squared successive difference (RMSSD)/min in % of the mean as the representation of variability per minute;

To better reflect the activity level, we expressed the SD and RMSSD as a percentage of mean activity counts per minute.

- Sample entropy as an indicator of the complexity of activity or level of regularity in the time series. A low sample entropy value indicates greater self-similarity or more regular time series, while a high sample entropy value indicates the opposite.

### Statistical analysis

We used SPSS (version 26) and Prism (version 9) for all statistical analyses. In the non-parametric analysis, statistical significance was set *a priori* at *p* < 0.05. Categorical variables were presented as frequencies and percentages and continuous variables as means (SDs).

For the demographic and clinical characteristics, we used the Kruskal–Wallis test to estimate the distribution of age, sex, years of education, and the total number of recording days between the groups.

In the between-group comparison, we calculated the mean activity level and SD for each parameter and adopted the Kruskal–Wallis test to perform a preliminary analysis of the difference in the morning and evening activity patterns between the groups. In addition, the Mann–Whitney U test was used for pairwise comparisons of the diagnostic groups, and the value of *p* was adjusted with Bonferroni correction for multiple tests. Changes in activity patterns between the groups were based on the mean activity level of each participant per minute for 24 h within 10 days, visualized using a three-dimensional graph, and examined using the Kruskal–Wallis test.

In the within-group comparison, we calculated each parameter and used the Wilcoxon signed-rank test to explore within-group differences in the activity patterns across the morning and evening periods. Based on the raw data of the mean activity counts per minute (morning and evening periods), a line graph and the Kruskal–Wallis test were used to further visualize and test the differences in the activity within each group, respectively.

## Results

### Demographic and clinical characteristics

The demographic and clinical characteristics of the sample are summarized in Table 1. A total of 30 patients were finally included in the study. Among them, 8 (26.7%) had BD-M (F31.1–F31.2: manic episodes with/without psychotic symptoms); 14 (46.7%), BD-D (F31.3–F31.5: acute depressive episodes with/without psychotic symptoms); and 8 (26.7%), BD-MS (F31.6: mixed episodes with/without psychotic symptoms). The mean age differed between the groups (BD-D: 20.71 [6.8] years vs. BD-M: 25.7 [6.76] years vs. BD-MS: 16.5 [3.34] years; *p* = 0.031). The three BD groups did not differ in the other demographic characteristics, including sex and years of education. Approximately 64.29, 75, and 62.5% of the patients in the BD-D, BD-M, and BD-MS groups were women, respectively. The total number of recording days did not differ between the groups (BD-D: 8.29 [2.73] days vs. BD-M: 9.00 [2.83] days vs. BD-MS: 9.75 [0.71] days; *p* = 0.322).

**Table 1:** Demographic and clinical characteristics.

	Mood states			Kruskal-Wallis test	
	BD-D (*N* = 14)	BD-M (*N* = 8)	BD-MS (*N* = 8)	*χ* ^2^	*p*
Age (Mean, SD)	20.71 (6.8)	25.75 (6.76)	16.5 (3.34)	6.965	0.031*
Female, *n* (%)	9 (64.29%)	6 (75%)	5 (62.5%)	0.395	0.821
Years of education (Mean, SD)	11.29 (2.64)	14.5 (2.93)	9.63 (1.51)	2.115	0.347
Self-injury, *n* (%)	6 (42.86%)	3 (37.5%)	8 (100%)		
Suicidal ideation, *n* (%)	12 (85.71%)	2 (25%)	8 (100%)		
Depressive symptoms, *n* (%)					
(1) mild			1 (12.5%)		
(2) moderate	6 (42.86%)		4 (50%)		
(3) severe	8 (57.14%)		3 (37.5%)		
Manic symptoms, *n* (%)					
(1) mild					
(2) moderate		2 (25%)	6 (75%)		
(3) severe		6 (75%)	2 (25%)		
Total days (SD)	8.29 (2.73)	9.00 (2.83)	9.75 (0.71)	2.267	0.322

*Post hoc* tests were adjusted by Bonferroni correction for multiple tests. BD-D, bipolar disorder-depression; BD-M, bipolar disorder-mania; BD-MS, bipolar disorder-mixed states. **p < 0.01, *p < 0.05.

The patients with BD-D were experiencing moderate (42.86%, *n* = 6) to severe (57.14%, *n* = 8) depressive symptoms. Most participants of the BD-M group were suffering from severe manic symptoms (75%, *n* = 6). In the BD-MS group, there were more patients with moderate depressive symptoms (50%, *n* = 4) and moderate manic symptoms (75%, *n* = 6). The medications used were different in subgroups and were shown in Table 2.

**Table 2:** Medication treatments for bipolar disorder subgroups.

Medication category	Mood states		
	BD-D (*N* = 14)	BD-M (*N* = 8)	BD-MS (*N* = 8)
Antipsychotics	14	7	7
Mood stabilizer	4	3	2
Antidepressants	3		1
Anxiolytics	2		

All values were displayed as *n*.

### Between-group comparison

**Figure 1:**
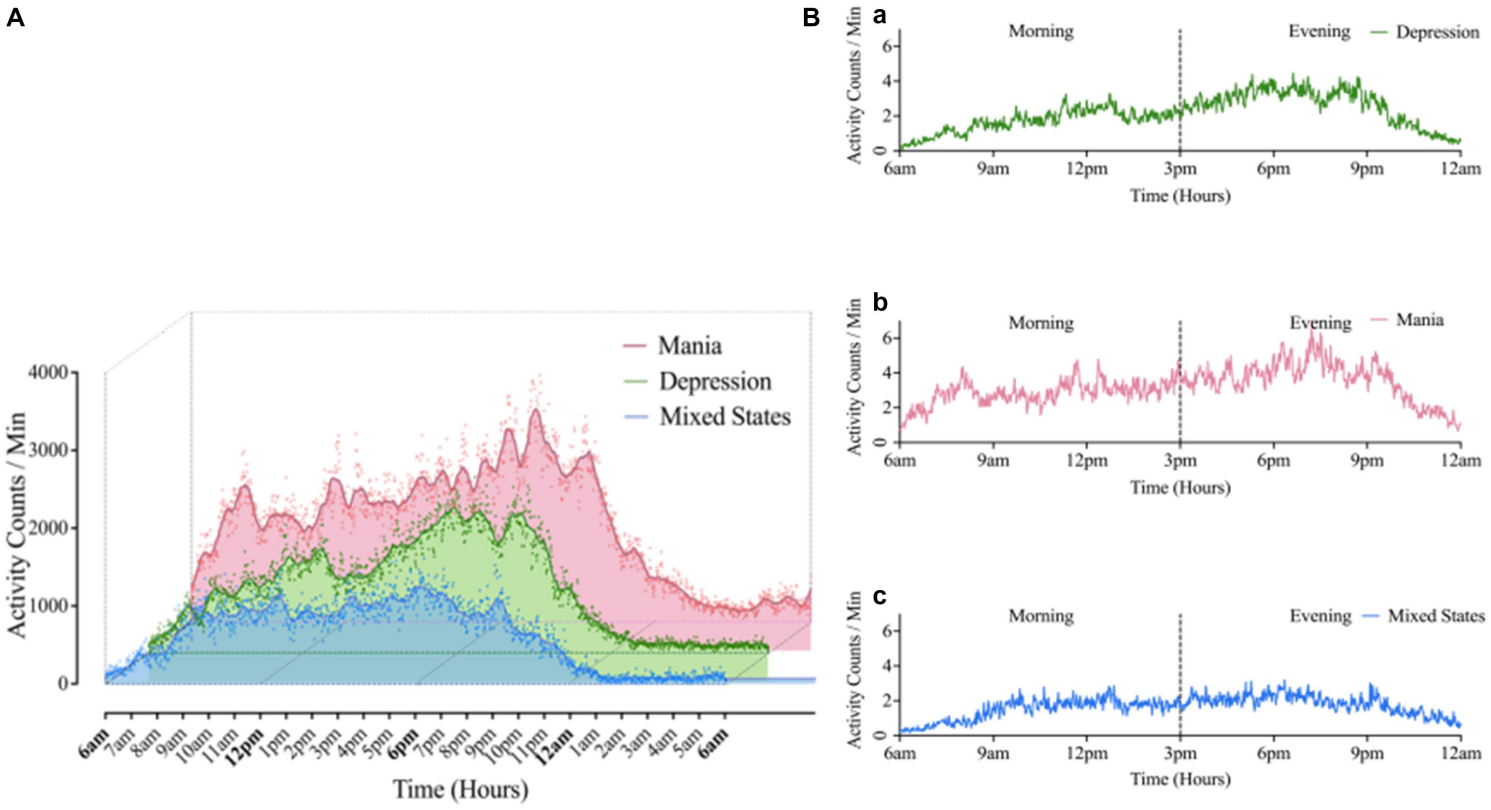
**(A)** Comparison of 24 h average motor activity in BD groups. The scatter represented the activity counts per minute, averaged over 10-days raw data for 24 h. **(B)** Morning-evening graph: Average 24 h motor activity during morning and evening periods in Mania (a), Depression (b), Mixed states (c) groups. Mean (Mania) = 1199.47; Mean (depression) = 851.86; Mean (Mixed States) = 641.02. “Kruskal-Wallis test showed there was significant difference in activity counts among three BD group, *χ*^2^ = 6.35, *p* = 0.042.”

As shown in Figure 1, the mean level of 24 h activity was significantly higher in the BD-M group than in the BD-D and BD-MS groups. In the separate analysis of the morning and evening periods (Table 3), we found that the average activity level in the morning did not significantly differ between the groups. However, both the SD/min in % of the mean of the intra-individual fluctuation (χ^2^ = 6.772, *p* = 0.034) and RMSSD/min in % of the mean of the minute-to-minute variability (χ^2^ = 7.522, *p* = 0.023) significantly differed. In the evening period, there were significant differences in both the mean activity level (χ^2^ = 8.197, *p* = 0.017) and minute-to-minute variability (RMSSD/min in % of the mean; χ^2^ = 8.956, *p* = 0.011). No significant differences in the sample entropy values were found in either the morning or evening period. The *post hoc* test demonstrated that in the evening period, the BD-M group showed a higher mean activity level (p = 0.017) and a lower RMSSD (expressed as a percentage of the mean activity counts per minute; *p* = 0.010) than did the BD-MS group. In the morning period, the BD-M group showed a lower SD (expressed as a percentage of the mean activity counts per minute; *p* = 0.047) and RMSSD (*p* = 0.024) than did the BD-MS group. No significant differences were observed in the *post hoc* test comparisons between the other groups.

**Table 3:** Comparisons of BD motor activity in between-group during morning and evening time perids.

Parameters	Period	BD-D	BD-M	BD-MS	*χ* ^2^	*p*	*Post Hoc* test
Mean	Morning	860.71	1363.3	728.93	5.383	0.068	
	Evening	1326.9	1648.9	915.82	8.197	0.017*	Mania group vs. Mixed-states group: *p* = 0.017
SD/min in % of mean	Morning	191.21	148.27	250.17	6.772	0.034*	Mania group versus Mixed-states group: *p* = 0.047
	Evening	148.57	141.71	201.17	4.211	0.122	
RMSSD/min in % of mean	Morning	160.09	122.05	177.39	7.522	0.023*	Mania group versus Mixed-states group: *p* = 0.024
	Evening	122.63	115.12	150.38	8.956	0.011*	Mania group versus Mixed-states group: *p* = 0.010
Sample entropy	Morning	0.304	0.377	0.298	0.159	0.924	
	Evening	0.403	0.237	0.345	3.172	0.205	

BD-D, bipolar disorder-depression; BD-M, bipolar disorder-mania; BD-MS, bipolar disorder-mixed states; SD, standard deviation; RMSSD, root mean square of successive difference. *Post hoc* tests were adjusted by Bonferroni correction for multiple tests. *p < 0.05.

### Within-group comparison

The Wilcoxon signed-rank test (Table 4) revealed that in the BD-D and BD-MS groups, the average activity level (BD-D: Z = −3.296, *p* = 0.001; BD-MS: Z = −2.100, *p* = 0.036), SD/min in % of the mean (BD-D: Z = −3.045, *p* = 0.002; BD-MS: Z = −2.380, *p* = 0.017), and RMSSD/min in % of the mean (BD-D: Z = −3.296, *p* = 0.001; BD-MS: Z = −2.240, *p* = 0.025) were higher in the evening than in the morning. In the BD-M group, we did not find similar changes but observed a significant decrease in the sample entropy values in the evening compared with those in the morning (BD-M: Z = 2.100, *p* = 0.036). As illustrated in Figure 1B, the BD-M group had an early-onset activity time. When the activity counts were assessed starting at 6 a.m., we found that the BD-M group had begun to have activity levels, while the BD-D and BD-MS groups were at an essentially zero activity level. As plotted in Figure 1A, smoothed data were based on the average 24 h activity level per minute of each participant across the monitoring period. Although the smoothing was approximated and not strictly Savitzky–Golay smoothing, we could observe multiple peaks in the motor activity map in the BD-M group and relatively smooth data in both the BD-D and BD-MS groups.

**Table 4:** Comparisons of BD motor activity between morning and evening time period in within-group.

Diagnosis	Morning vs. evening period	Wilcoxon signed-rank test		
	(Activity count per minute)	Difference	*Z*	*p*
BD-D	Mean	−466.164	−3.296	0.001**
	SD in % of mean activity count	42.639	−3.045	0.002**
	RMSSD in % of mean activity count	37.46	−3.296	0.001**
	Sample entropy	−0.099	1.412	0.158
BD-M	Mean	−285.64	−1.400	0.161
	SD in % of mean activity count	6.556	−0.420	0.674
	RMSSD in % of mean activity count	6.93	−1.120	0.263
	Sample entropy	0.140	−2.100	0.036*
BD-MS	Mean	−186.886	−2.100	0.036*
	SD in % of mean activity count	48.999	−2.380	0.017*
	RMSSD in % of mean activity count	27.008	−2.240	0.025*
	Sample entropy	−0.047	0.000	1.00

BD-D, bipolar disorder-depression; BD-M, bipolar disorder-mania; BD-MS, bipolar disorder-mixed states; SD, standard deviation; RMSSD, root mean square of successive difference. **p < 0.01, *p < 0.05.

## Discussion

In this study, we monitored more complete 24 h activity cycles during acute episodes of BD. During the relatively long monitoring period, the BD-M group had higher average activity levels than the BD-D and BD-MS groups and showed a trend toward more frequent activity. Similar to other studies that monitored motor activity within 24 h of acute admission, the manic group had a higher average activity level than other groups (29, 30). At the behavioral level, different combinations in neurotransmitter transmission tend to balance the network between different functional brain states, driving into either excitation in psychomotor activity (facilitating motor activity and impulsivity), or inhibition in psychomotor activity (inhibiting sensory responsivity and delaying motor responses) (31). Although the total average activity levels could not be used to utterly distinguish the three groups, the average activity levels between the BD-M and BD-MS groups in our study differed, but not between the BD-M and BD-D groups. The reasons may be that the sample size was small, and the closed management of the wards during the epidemic of COVID-19 led to regular hospitalization and reduced activity areas, which limited the diversity of activities; these factors may have reduced the differences between the groups.

In the morning period, there were significant differences in the intra-individual fluctuations and variabilities per minute between the groups. Both parameters increased in the BD-D and BD-MS groups compared with those in the BD-M group. Our findings are partially consistent with previous results during specific active morning and evening periods. A previous study of continuous active 64-min morning activity reported similar differences (11). Herein, the patients with BD-D had significantly increased intra-individual fluctuations and minute-to-minute variability compared with those with BD-M. A recent study demonstrated that the activity patterns were comparable between hospitalized patients with mixed state and mania. However, we found a more pronounced difference between the BD-MS and BD-M groups, the reason for which may be that our study covered longer and more complete periods. We also made a strict diagnosis in accordance with the ICD-10 that the symptoms of mania and depression were equally prominent in the past 2 weeks; however, the severity of each symptom was not as detailed as that in previous studies (32). Another reason was the difference in the composition of the groups and the number of female patients included.

In the evening period, we found that the BD-M group had a higher average activity level but a smaller minute-to-minute variability than the other groups. The intra-individual fluctuations differed between the groups, but pairwise comparison did not reveal any significant findings. BD-M is generally characterized by psychomotor excitement and agitation. This clinical presentation can be explained by the abnormally increased neuronal variability in the somatomotor network (33–35). In the different states of BD, the regional distribution of neuronal activity is abnormally altered. However, it remains unclear how the global brain activity assessed on global signal topography changes during a particular phase of BD. Without limitations of the treatment schedule, the higher the autonomous motor activity levels in the evening, the greater the features of BD-M activity. The small variability in the BD-M group is consistent with the actigraphic data reported by (27).

Interestingly, the BD-MS group showed a similar tendency to the BD-D group in both morning and evening periods. Barroilhet et al. showed that psychomotor activation was a core symptom of the mixed state and was unrelated to polarity (36). Thus, psychomotor activation had an essential involvement. Except for the increasing frequency of thoughts, contacts, and goal-directed activities usually reported, the mixed state can sometimes manifest as slowed or inhibited thoughts and motor activities unrelated to depressive moods (37, 38). This manifestation was described by Kraepelin as the inhibited manic subtype. However, (11) reported that the mixed state is similar to mania. The mixed state used to be a mood state of BD I; its diagnosis has recently been transferred into a mixed-feature specifier for manic/hypomanic and depressive episodes in the DSM-5 (American Psychiatric Association, 2013). As a result of this transition, there is potential for more individuals with the mixed state to be recognized and diagnosed. Mood states can be conceptualized on a continuum. The mixed state can be seen as a transitional state. At the extremes are pure mania and pure depression, and in between are a variety of mixed states (39). Combining previous studies with our findings, the patients in the mixed state should not be arbitrarily incorporated into the manic or depressive group. The motor activity of BD-MS patients may be variable. Our findings suggest that the activity parameters of patients with the mixed-feature specifier for different episodes should be analyzed with larger sample sizes.

The intra-day activity variation may reflect the variation in mood changes and identify different mood states (40). Our study found that the BD-M, BD-D, and BD-MS groups all showed a trend toward increased mean activity counts from morning to evening, consistent with some but not all discoveries. This trend may imply that the activity patterns have similarities between different patients with BD. Analogous trends were observed in previous studies: Patients with BD-M and BD-MS mostly showed an increasing trend, while patients with BD-D showed a decreasing trend (11, 28). The possible reason for this discrepancy could be that our study included both the morning and evening periods in comparison with specific consecutive active periods. The complete cycle of activity monitoring allowed the data to be more comprehensive. Similarly, such monitoring was more susceptible to the scheduled inpatient treatment. The majority of the physical therapy activities of the patients were concentrated in the morning. To some extent, our ability to differentiate the motor activity patterns between the patients with the mixed state, depression, and mania was restricted. This explained the varying activity trends between our study and other research.

Herein, the BD-D group had significant changes in the intra-day activity. From morning to evening, the intra-individual fluctuations and minute-to-minute variability decreased, while the mean activity level increased. The BD-MS group showed the same changing trend as the BD-D group. Based on the activity images, the BD-D and BD-MS groups had reduced, and more variable activity and a delayed morning activity onset compared with the BD-M group, consistent with previous reports (5, 27, 41). The changing trend in the patients with BD-D and BD-MS was partially attributed to the fact that the activities in the evening were freely scheduled without external interference. In contrast, a fixed treatment time in the morning could result in a sudden decrease in activity and lead to greater intra-individual and minute-to-minute variabilities.

Reduced differences in morning and evening activity levels; and reduced activity complexity (decreased sample entropy values) at night were found in the BD-M group. This group was active throughout the day, and we did not find any significant differences in the intra-day activity variation, except for the sample entropy value. The sample entropy value of the BD-M group was higher in the morning, suggesting less self-similarity and more irregular time series in the morning among patients with mania. The small variability and high entropy value were regulated in a previous specific short-time monitoring study (11). In our research, the BD-M group was treated in the morning but showed changes different from those of the BD-D and BD-MS groups. This possibly indicates that the disorganized activity of patients with BD-M persists throughout the day even under the influence of a scheduled treatment. Thus, the variation among patients with BD-M from morning to evening did not reflect an intra-day change. The rhythm in these patients is non-negligible. Our findings may contribute to a better understanding of the intra-day variation in patients with BD.

There are some notable limitations of this study. First, although our study somewhat extended the follow-up period, it failed to thoroughly observe the variation from acute onset to remission in each mood state. This may account for the inability to distinguish between the mood groups. Second, the small sample of patients with BD observed in a limited activity space may influence the results. Accordingly, the results should be interpreted with caution. A similar schedule arrangement during hospitalization may also affect the activities of patients. As we mentioned above, the relatively strict rules and routines of the enclosed wards, as well as limited activity space, could equalize physical activity patterns. Moreover, this study did not include age as a covariate to further discuss the effect on motor activity. The mean age was low in all three groups, although there is robust evidence in recent decades that the peak age range for adult-pattern BD onset is approximately 15–25 years (42). However, our proof-of-concept study with a relatively small sample size prevented us from further stratifying and left us without age-appropriate controls. To further investigate the generalizability and accuracy of the motor activity findings, future studies should expand the sample size and conduct a controlled study. Third, actigraphy monitoring in our study was performed only in spring and summer. Therefore, results may be affected by seasons. In a review of previous studies, Rosenthal et al. (43) proposed that seasonal variations in photoperiod were associated with the onset and severity of BD symptoms and hospitalizations. And human circadian rhythms varied according to the seasonal changes in photoperiod, with increased motor activity in the spring and decreased activity in the autumn. Similarly, Goodwin and Jamison (44) found that manic/hypomanic symptoms peaked in the spring and early summer and that suicide attempts and completions were high in the spring and early summer (45) while depressive symptoms peaked in the fall and winter. Besides expanding the sample size, future studies should repeat the measurements across multiple seasons. Finally, different drug treatments and other potential confounding factors, such as the severity of current episodes, body mass index, and comorbidities, may affect motor activity (46). These factors may influence the accuracy of the differences observed in the activity between the groups.

Despite these limitations, our study objectively and comprehensively analyzed different states of BD within a longer period. In the analyses, we observed the motor activity of the BD-MS group showed a similar tendency to that of BD-D group and the disorganized activity of patients with BD-M persists throughout the day. Our findings provide preliminary evidence that there are differences and similarities in the activity patterns across the three mood states. Therefore, further investigation is needed on digital biomarkers that can discriminate between mood states or refine the diagnosis. Nevertheless, these findings are beneficial in supplementing the traditional diagnosis of BD. The existing assessment of psychiatric diagnosis relies heavily on clinicians, which is time-consuming and labor-intensive (47). We used a non-invasive digital technology, actigraphy, to collect biometric parameters in a daily environment. Our study demonstrated the feasibility of objective and non-invasive recording of long-term motor activity parameters in BD patients during acute episodes. With the development of such a digital biomarker, an exact correspondence between mood episodes and activity patterns may be found. Ongoing activity monitoring may clarify the current emotional state of patients. This may aid in recognizing the onset of depression, mania, or the mixed state of BD and in providing individualized treatment and care plans according to different episodes.

## Data availability statement

The raw data supporting the conclusions of this article will be made available by the authors, without undue reservation.

## Ethics statement

The studies involving human participants were reviewed and approved by the ethical committee of the First Affiliated Hospital of Chongqing Medical University. Written informed consent to participate in this study was provided by the participants’ legal guardian/next of kin.

## Author contributions

QL, XL, YZ, and XD: study design. QL and XL: study guidance. QL, YZ, and HL: patient sceening and evaluation. YZ, XW, and HL: data collection. XL, XD, and YZ: analysis and interpretation of data. YZ: drafting of original manuscript. QL, XL, XD, and HL: critical revision of the manuscript. All authors contributed to the article and approved the submitted version.

## Funding

This research was supported by Chongqing medical scientific research project (Joint project of Chongqing Health Commission and Science and Technology Bureau; no. 2020GDRC026) and the National Key Research and Development Program of China (no. 2021YFC2501500). The sponsors of the study had no role in study design, data collection, analysis, and interpretation, or the manuscript writing.

## Conflict of interest

The authors declare that the research was conducted in the absence of any commercial or financial relationships that could be construed as a potential conflict of interest.

## Publisher’s note

All claims expressed in this article are solely those of the authors and do not necessarily represent those of their affiliated organizations, or those of the publisher, the editors and the reviewers. Any product that may be evaluated in this article, or claim that may be made by its manufacturer, is not guaranteed or endorsed by the publisher.
